# BACTERIOLOGICAL STUDY OF AEROBIC ISOLATES FROM PLANTAR ULCERS OF PAUCIBACILLARY LEPROSY PATIENTS

**DOI:** 10.4103/0019-5154.60350

**Published:** 2010

**Authors:** Monalisa Majumdar, Urmita Chakraborty, Jayasri Das, Jayashree Nath Barbhuiya, Gautam Mazumdar, Nishith Kumar Pal

**Affiliations:** *From the Department of Microbiology, Calcutta School of Tropical Medicine, Kolkata, India.*; 1 *From the Department of Dermatology, Calcutta School of Tropical Medicine, Kolkata, India.*

**Keywords:** *Leprosy*, *plantar ulcer*, *bacteria*

## Abstract

**Background:**

Plantar ulcers commonly occur in leprosy patients, which usually recur and cause morbidity in such cases.

**Aims:**

The aim of the study is to find out the bacteriological profile of these ulcers and to find out the antibiotic susceptibility of the isolates so that appropriate drugs may be chosen for treatment and for prevention of recurrence.

**Materials and Methods:**

Fifty-six samples from recurrent plantar ulcers of paucibacillary leprosy patients (attending the outpatient department of Calcutta School of Tropical Medicine) were studied for the purpose. Proper sample collection, gram staining, inoculation on culture media, and final identification by biochemical methods were undertaken. Antibiotic susceptibility testing was done for appropriate choice of drugs.

**Results:**

Mixed growth of bacteria was seen in 20 (36%) cases while single organism was isolated from the rest. *Staphylococcus aureus* is the predominant single isolate followed by *E. coil, Proteus sp*. and *Pseudomonas sp*. Chloramphenicol and gentamycin are the two drugs that have shown efficacy to the extent of 75 to 100% and 25 to 100% respectively *in vitro* studies.

**Conclusion:**

Bacteriological study of plantar ulcers of leprosy patients has revealed *Staphylococcus aureus* as the main pathogen. Treatment with chloramphenicol and gentamycin holds good prospect as per our study.

## Introduction

Plantar ulceration is one of the most common complications for which leprosy patients seek treatment. These ulcers are also known as trophic ulcers. Plantar ulcers, as the name implies, occur on the sole of the foot, especially in the fore part of the sole (ball of the foot) where 70 to 90% of ulcers are located. Failure of treatment, chronicity and recurrence of these plantar ulcers in poor leprosy population cause economic losses to the families. The foot with sensory loss is prone to develop ulcers due to cracks and fissures and trauma from external sources and also due to internal injury caused by walking and other activities of the foot.[1] Surface wounds are often colonized with environmental bacteria and swab samples taken from surface sometimes do not depict the real cause of the infectious process.

The present study has been undertaken in The School of Tropical Medicine, Kolkata, to evaluate the causative organisms of these ulcers and to find out remedial measures to lessen morbidity in such cases.

## Materials and Methods

### Case selection

Thirty two males and 24 females (ages varying from 20 to 40 yr) suffering from leprosy, each with a single plantar ulcer on the ball of foot were selected for our study. All were tested negative for slit skin smear for acid-fast bacilli with modified Ziehl-Neelsen staining for *Mycobacterium leprae*.

The plantar ulcers were chronic, indolent, with scanty discharge and a pale, unhealthy fibrosed base.

### Collection of materials

The superficial part of the ulcer was cleaned with spirit and iodine, the slough was removed and samples were taken from the depth of the ulcer with sterile bacteriological loop after allowing the iodine to remain for half an hour.

### Identification

The swabs were processed for gram stain and culture. For isolation of the aerobes, inoculation was done on nutrient agar, blood agar and MacConkey's agar media and incubated overnight at 37°C. Identification of the isolates were done using biochemical methods.[2] The isolates were further tested for antibiotic sensitivity on Mueller Hinton agar medium using Kirby Bauer technique (using discs of Hi Media) following National Commmittee for Clinical Laboratory Standards (NCCLS) guideline.[3]

## Results

Out of 56 samples studied, aerobic bacterial growth was noted in 54 cases. Regarding the distribution of bacteriological profile of aerobic organisms, *S. aureus, E. coil, Proteus sp.* and *Pseudomonas sp.* were isolated in 32, 16, 22 and 4 cases respectively. No growth of organism was found in two cases. Mixed growth (more than one organism) was noticed in 20 (36%) samples [Table 1]. In 34 cases, one type of bacteria in pure culture was noted. Genus and species level of their distribution is given in Table 2. Antibiogram studies were conducted only with the pure single isolates as shown in Table 3.

**Table 1:** Pattern of mixed growth

Growth	No. (%)
Mixed growth of bacteria	20 (36)
*Staphylococcus aureus* and *Proteus sp*.	12 (60)
*Proteus sp.* and *E. coli*.	5 (25)
*Proteus sp*. and *Pseudomonas sp*.	1 (5)
*Staphylococcus aureus* and *E. coli*	2 (10)

**Table 2:** Distribution of pure single isolates (*n* = 34)

Organism	No. of cases	Percentage of pure isolate
*Staphylococcus aureus*	18	53
*Escherichia coli*	9	26
*Proteus sp.*	4	12
*Pseudomonas sp.*	3	9

**Table 3:** The drug sensitivity pattern of the pure isolates

Antibiotics (Disc content in μg)	No. of sensitive isolates (%)			
	
	*S. aureus* (18)	*E. coli* (9)	*Proteus* (4)	*Pseudomonas sp.* (3)
Vancomycin (30)	18 (100)	-	-	-
Ciprofloxacin (5)	7 (39)	8 (89)	2 (50)	2 (67)
Gentamycin (120)	14 (78)	7 (78)	1 (25)	3 (100)
Chloramphenicol (30)	14 (78)	8 (89)	3 (75)	3 (100)
Gatifloxacin (5)	14 (78)	8 (89)	2 (50)	2 (67)

## Discussion

Plantar ulcers are prone to soiling, especially in people observing poor hygiene and often walking without proper shoes as required by leprosy cases. Attempt to isolate the causative organisms is a constraint due to the fact that most of the time the ulcer surface is contaminated and colonized by environmental bacteria. It is very difficult to collect materials from the depth of the ulcers for the collection of suitable materials for the study of bacteriological profile in such cases. However, surface cultures have been practiced by earlier investigators.[4] This view is corroborated by the study of Ebenezer *et al.*, who showed *Proteus sp*. as the dominant organism, followed by *E. coli*.[5] In our study, *S. aureus* has emerged as the predominant isolate followed by *E. coli*. This variance might be due to the effort in our study to collect representative sample from the depth of the ulcer, avoiding superficial slough and dirt, usually colonized by environmental bacteria. Still the present study revealed mixed growth in 20 (36%) cases. This indicates either inadequacy of sample collection process, or polymicrobial infection in such cases. As we could not establish the answer to this question, antimicrobial susceptibility test was done for pure single isolates only.

The recent study shows that *Staphylococcus aureus*, the most virulent of all Staphylococci encountered, is the most prevalent among the aerobic isolates from these plantar ulcers. The invasive nature of the organism poses a threat for deeper tissue invasion and bacteriemia. *In vitro* studies show that chloramphenicol and gentamycin are the two drugs that have efficacy to the extent of 75 to 100 and 25 to 100% respectively.

Plantar ulcers often lead to morbidity and/or poor quality of life of leprosy patients. If remedial measures are not enforced urgently, crippling conditions will become a permanent handicap for them. Infective conditions, such as osteomyelitis, septic arthritis and septic tenosynovitis due to spread of infection to underlying bones, joints and tendon sheaths, are the most common complicating features found in complicated ulcers.[6]
